# Deguelin Induces Apoptosis by Targeting Both EGFR-Akt and IGF1R-Akt Pathways in Head and Neck Squamous Cell Cancer Cell Lines

**DOI:** 10.1155/2015/657179

**Published:** 2015-05-17

**Authors:** Yuh Baba, Masato Fujii, Toyonobu Maeda, Atsuko Suzuki, Satoshi Yuzawa, Yasumasa Kato

**Affiliations:** ^1^Department of General Clinical Medicine, Ohu University School of Dentistry, 31-1 Mitsumido, Tomiya-machi, Koriyama City, Fukushima 963-8611, Japan; ^2^National Institute of Sensory Organs, National Tokyo Medical Center, 2-5-1 Higashigaoka, Meguro, Tokyo 152-8902, Japan; ^3^Department of Oral Function and Molecular Biology, Ohu University School of Dentistry, 31-1 Mitsumido, Tomiya-machi, Koriyama City, Fukushima 963-8611, Japan

## Abstract

Deguelin, a rotenoid compound from the African plant *Mundulea sericea* (Leguminosae), has been shown to possess antitumor activities but the exact role for the growth factor receptor mediated signaling pathway in head and neck squamous cell carcinoma (HNSCC) is currently still unclear. In the present study, we investigated the effect of deguelin on epidermal growth factor receptor (EGFR) and insulin-like growth factor-1 receptor (IGF1R) pathways in HNSCC cell lines. Flowcytometric analysis revealed accumulation of annexin V positivity in deguelin-treated cells, showing that deguelin induced apoptosis. The deguelin-induced apoptosis was accompanied by the reduction of constitutive phosphorylated levels of IGF1R, Akt, and extracellular signal-regulated kinase1/2 (ERK1/2). LY294002-mediated inhibition of phosphatidylinositol-3 kinase, which is an upstream effector for Akt activation, increased cleavage of poly(ADP-ribosyl) polymerase (PARP) but ERK inhibition by U0126 did not. Deguelin inhibited both IGF-1- and EGF-induced Akt activation. These results showed that deguelin possessed antitumor effect by targeting Akt in dual axis such as EGFR and IGF1R signaling pathways and suggested that it provides an applicable therapeutic strategy for HNSCC patients.

## 1. Introduction

Head and neck squamous cell carcinoma (HNSCC) is the sixth most common neoplasm worldwide, with approximately 600,000 patients newly diagnosed each year [1]. Over the past 30 years, patients with recurrent and/or metastatic HNSCC have had a poor prognosis [2, 3]. A total of 30–50% of patients develop local or regional recurrence, with more patients developing distant metastases [4, 5]. Therefore, research focused on gaining a better understanding of this disease and on the development of novel treatment strategies is required.

Epidermal growth factor receptor (EGFR) is a ubiquitously expressed transmembrane glycoprotein belonging to the ErbB/HER family of receptor tyrosine kinases (TK). Activation of EGFR leads to autophosphorylation and activation of intracellular signaling pathways including the phosphatidylinositol 3-kinase (PI3K)/Akt pathway (as a survival signal) and extracellular signal-regulated kinase 1/2 (ERK1/2) pathway (as a proliferation signal). EGFR is abundantly expressed in squamous cell carcinomas including head and neck region [6]. Because elevated expression of EGFR in HNSCC correlates with poor prognosis and EGFR plays critical roles in cell survival and proliferation, EGFR signaling had been thought to be the most important target as the anticancer treatment strategy [7]. Therefore, the use of EGFR inhibitors such as gefitinib and erlotinib was expected to be applicable strategy for HNSCC therapy. However, clinical study showed disappointing results; that is, respective overall response rate for gefitinib and erlotinib was 11% [8] and 4% [9] in the patients with recurrent and/or metastatic HNSCC. As we have previously postulated that crosstalk between EGFR-Akt and IGF1R-Akt pathways is thought of as one mechanism of low response rate of EGFR inhibitor alone for HNSCC patients [10], management for both signaling pathways should be considered for the patients with HNSCC.

Deguelin, which is a rotenoid isolated from the African plant* Mundulea sericea *(Leguminosae), is a potent chemopreventive agent for some kinds of cancers. Using it in mouse chemical carcinogenesis assay, it has been shown that deguelin suppresses formation of not only aberrant crypt foci in colons [11], skin papilloma [12, 13], and lung tumor [14] but also carcinoma formation such as mammary grand adenocarcinoma [13].

In recent years, molecular mechanism of deguelin's function has been uncovered. Many functions of deguelin have been reported by Yang et al. [15]; that is, deguelin has an inhibitory activity for Akt signaling, and deguelin disrupts association between heat shock protein (HSP) 90 with survivin and cyclin-dependent kinase 4, while inducing ubiquitination followed by the degradation. They also reported that deguelin induces ceramide production which results in apoptosis by autophagy through the ceramide-AMP-activated protein kinase-Ulk1 axis [15]. Although deguelin could be reduced by both EGFR-Akt [16] and IGF1R-Akt pathways [17] in breast cancer model, the potential effect of deguelin on those pathways in HNSCC is still unknown. Therefore, we determined whether deguelin has inhibitory activity for both EGFR-Akt and IGF1R-Akt pathways to induce apoptosis in HNSCC.

## 2. Methods

### 2.1. Reagents

**Figure 1:**
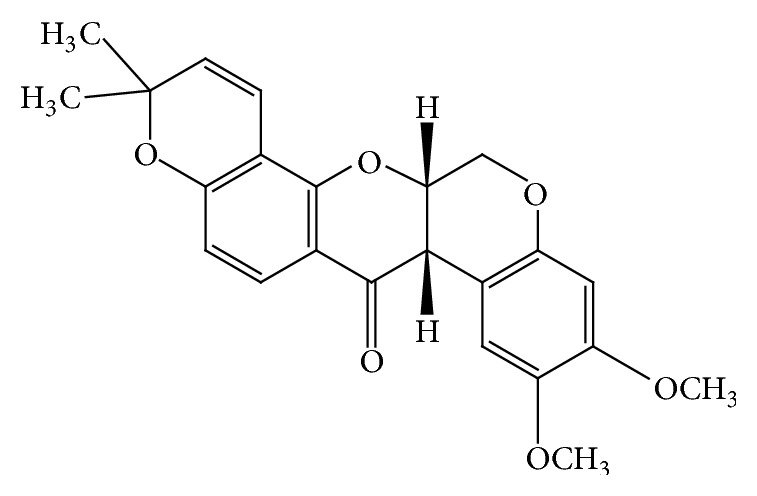
The chemical structure of deguelin.

Dulbecco's modified Eagle's medium (DMEM) was from Nissui (Tokyo, Japan). Fetal bovine serum (FBS) was from Hyclone (South Logan, UT, USA). Deguelin (Figure 1), purchased from Wako (Osaka, Japan), was dissolved in DMSO as a 50 mM stock solution, stored as small aliquots at −20°C. U0126 (ERK kinase (MEK) inhibitor), LY294002 (phosphatidylinositol 3-kinase (PI3K) inhibitor), and rabbit monoclonal antibodies against p-Akt (Ser^73^), total-p44/42 MAPK (ERK1/2), total-IGF1R, and phosphorylated-EGFR (p-EGFR; Tyr^1068^) and rabbit polyclonal antibodies against total-Akt and poly(ADP-ribosyl) polymerase (PARP) were purchased from Cell Signaling Technology (Beverly, MA, USA). Rabbit polyclonal antibodies against glyceraldehyde 3-phosphate dehydrogenase (GAPDH) were from GeneTex (Irvine, CA, USA). Mouse monoclonal antibody against phosphorylated-ERK1/2 (p-ERK1/2) and rabbit polyclonal antibody against total EGFR were from Santa Cruz Biotechnology (Dallas, TX, USA). Rabbit recombinant oligoclonal antibodies against phosphorylated IGF1R (p-IGF1R; Tyr^1135^/Tyr^1136^) were from Invitrogen (Carlsbad, CA, USA), and mouse monoclonal antibody against p-EGFR (Tyr^1173^) was from Millipore (Billerica, MA, USA). Anti-annexin V antibody, conjugated with a fluoroisothiocynate fluorescence dye, was from Bio-Rad (Hercules, CA, USA). Biotin-conjugated goat anti-mouse IgG (H+L) and biotin-conjugated goat anti-rabbit IgG (H+L) were from Jackson ImmunoResearch (West Grove, PA, USA). Blocking Reagent N102 was from NOF Corp. (Tokyo, Japan). Chemiluminescence reagent was from Amersham (Buckinghamshire, UK). Protein assay kit was from Bio-Rad. Bovine serum albumin was from Sigma-Aldrich (St. Louis, MO, USA).

### 2.2. Cell Lines and Culture

SCC-4 cells and HSC-4 cells, cell lines derived from human tongue carcinoma, were provided from the Human Science Research Resources Bank (HSRRB) (Osaka, Japan). They were maintained in DMEM supplemented with 10% FBS and 100 U/ml penicillin G and 100 *μ*g/ml streptomycin. Cells were incubated at 37°C in a humidified atmosphere containing 5% CO_2_ and 95% air.

### 2.3. Cell Viability Assay

SCC-4 cells (2 × 10^5^ cells/ml) and HSC-4 cells (1 × 10^6^ cells/ml) were cultured in complete DMEM medium in the presence of 0 and 100 *μ*M deguelin in 6-well tissue-culture plate (Thermo Fisher Scientific, Hudson, NH, USA). After 24 h of culture, the cell numbers were determined by the trypan-blue dye exclusion method.

### 2.4. Analysis of Cell Cycle

After incubation period, cells were collected by the trypsin treatment and fixed with 70% ethanol. The cellular DNA was stained for 30 min with 0.1 mg/ml propidium iodide solution. Finally, the cells were analyzed via flow cytometry (Epics Elite, Coulter, Hialeah, FL, USA).

### 2.5. Annexin V Assay

To identify apoptosis, we detected annexin V positivity by flow cytometry. Cells (5 × 10^5^) were incubated with 100 *μ*M deguelin and then stained. They were washed twice in PBS, resuspended in 100 *μ*L of a binding buffer containing a fluoroisothiocynate-conjugated anti-annexin V antibody and propidium iodide, and then analyzed by the flow cytometry (FACS Calibur; BD Biosciences, San Jose, CA, USA).

### 2.6. Western Blot Analysis

Protein level was compared by Western blot analysis which was described elsewhere [18]. In brief, proteins in whole-cell lysates were electrophoresed on sodium dodecyl sulfate containing 7.5% polyacrylamide gel and they were electrotransferred onto polyvinylidene fluoride (PVDF) membranes. After blocking with 20% Blocking Reagent N102, the membrane was treated with first antibody of interest, followed by treatment with biotin-conjugated secondary antibody. Signals were detected with chemiluminescence reagent. The blots were stripped and reprobed with anti-GAPDH antibodies to show equal protein loading. Intensity of immunoreacted bands was quantified by Scion Image (Scion Corp., Frederick, MD, USA).

### 2.7. Protein Assay

The protein content in the lysates was measured according to Lowry method using Bio-Rad protein assay kit with bovine serum albumin as the standard.

### 2.8. Statistical Analysis

Statistical significance was calculated using Student's *t*-test. *P* values less than 0.05 were considered significant.

## 3. Results

### 3.1. Deguelin Induced Cell Death in SCC-4 and HSC-4 Cell Lines

**Figure 2:**
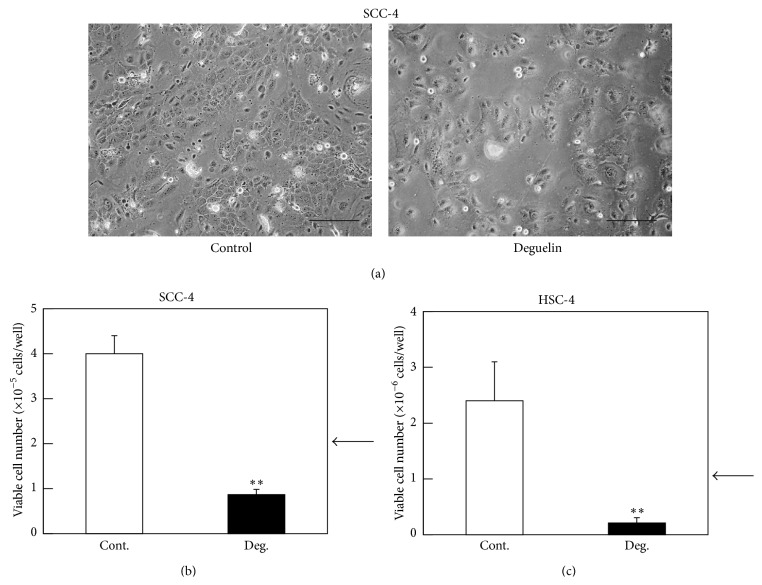
Deguelin induced cell death in SCC-4 and HSC-4 cell lines. Phase-contrast microscopic analysis. SCC-4 cells were treated with 0 or 100 *μ*M deguelin in DMEM + 10% FBS. After 24 h incubation, photographs were taken under phase-contrast microscopy. Representative phase-contrast micrographs are shown (a). Bar: 50 *μ*m. Trypan-blue dye exclusion assay was performed to measure cell viability of SCC-4 cells (b) and HSC-4 cells (c) at 24 h after 100 *μ*M deguelin treatment. Arrows indicate initial cell numbers. Each point represents the mean ± SD from triplicate assay (^∗∗^ *P* < 0.01).

We examined whether deguelin suppresses the proliferation of human tongue squamous cell carcinoma cell lines, using trypan blue dye exclusion method. As shown in Figure 2, deguelin treatment inhibited proliferation of SCC-4 and HSC-4 cells. Viable cell numbers after deguelin treatment were less than initial cell numbers (Figures 2(b) and 2(c)), suggesting that deguelin induced cell death in both SCC-4 and HSC-4 cell lines.

### 3.2. Deguelin Induced Apoptosis

**Figure 3:**
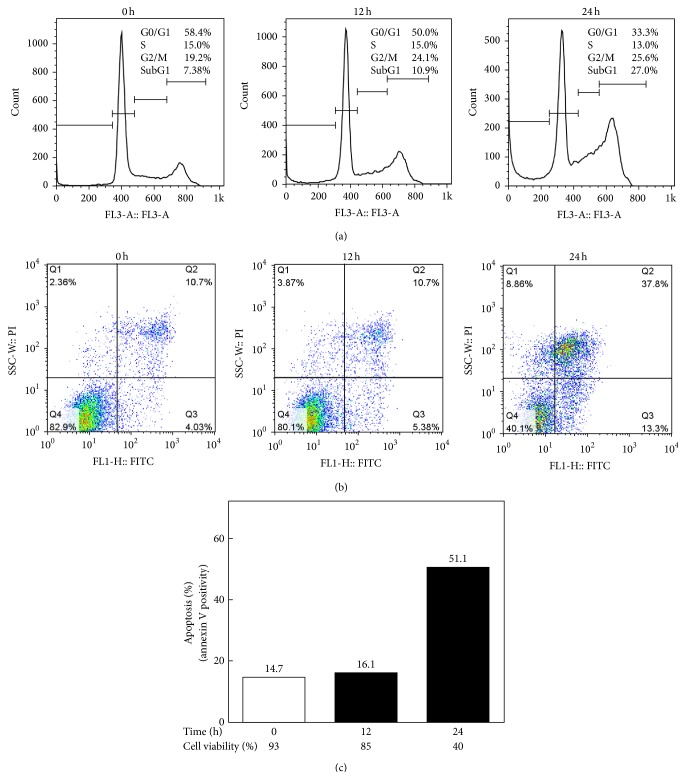
Deguelin induced apoptosis in SCC-4 cell lines. SCC-4 cells were incubated in the absence or presence of 100 *μ*M deguelin for different times. Thereafter, the cells were washed and fixed. They were further stained with propidium iodide (PI, *x*-axis) to detect accumulation of cell cycle phase (a) and treated with anti-annexin V antibody conjugated with FITC (FITC, *y*-axis) to analyze apoptosis (b) by flow cytometry.

Cell cycle analysis was performed using flow cytometry. Deguelin-treated SCC-4 cells accumulated in the sub G1 phase (27.0%) by 24 h treatment as compared with its vehicle control (7.38%) (Figure 3(a)). Then, annexin V positivity in deguelin-treated cells was evaluated using flow cytometric analysis (Figures 3(b) and 3(c)). Deguelin-induced apoptotic cell population in early stage (annexin V^+^/propidium iodide^−^) increased to 13.30% from 4.03% (basal level) after 24 h treatment while those in late stage (annexin V^+^/propidium iodide^+^) reached 37.8% from 10.7% (basal level) after 24 h treatment. Overall apoptotic cell population by deguelin was increased from 14.7% to 51.1% in a time-dependent manner.

### 3.3. Deguelin Reduced the Expression of p-IGF1R, p-Akt, and p-ERK

**Figure 4:**
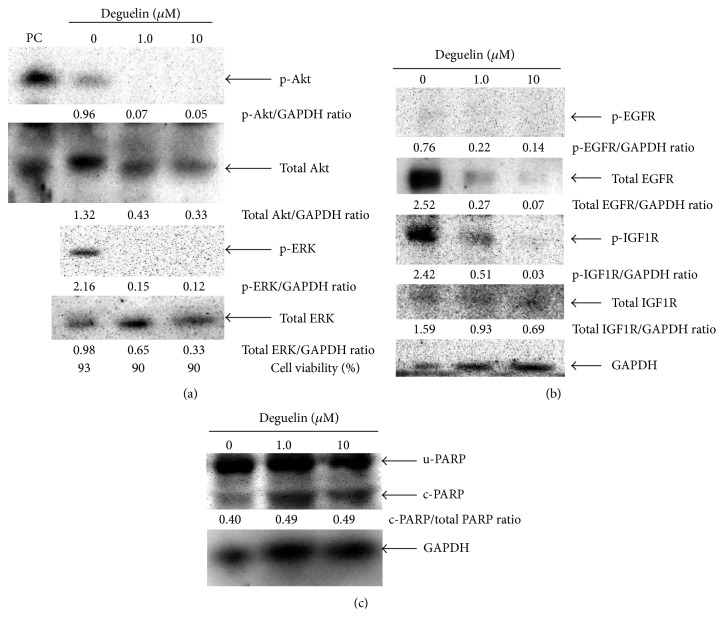
Deguelin reduced the expression of phosphorylated IGF1R, p-Akt, and p-ERK and induced apoptosis in SCC-4 cell lines. Subconfluent culture was treated with deguelin at different concentrations for 24 h. Whole-cell extracts were prepared and analyzed by Western blot using antibodies against p-Akt, Akt, p-ERK, and ERK (a); p-EGFR, EGFR, p-IGF1R, and IGF1R (b); and PARP (c-PARP, cleaved PARP; u-PARP, uncleaved PARP; total PARP, sum of cleaved and uncleaved PARP) (c). Total cell extracts from Jurkat cells: serum starved overnight and then treated with Calyculin A was used as positive control (PC) for p-Akt and Akt.

The majority of the HNSCC cells show overexpression of EGFR, whose activation leads to activation of intracellular signaling including the PI3K/Akt and ERK pathways. Although deguelin has been shown to inhibit Akt activation, the effect of deguelin on EGFR signaling cascade is still not known in HNSCC. As shown in Figure 4, deguelin reduced the expression of total EGFR, p-Akt, and p-ERK in SCC-4 cells. We could not detect constitutive level of p-EGFR in the standard culture condition, suggesting that Akt and ERK are not a downstream target of EGFR but possibly IGF1R which was examined later. Expectedly, IGF1R has been constitutively phosphorylated as the basal level and deguelin reduced its phosphorylation concordance with the elevation of PARP cleavage (Figures 4(b) and 4(c)). These results suggested that deguelin induced apoptosis with the suppression of both IGF1R-Akt and IGF1R-ERK pathways.

### 3.4. Deguelin-Induced Downregulation of p-IGF1R, p-Akt, and p-ERK Is Not due to Its Effects on Cell Viability

To exclude the possibility that the downregulation of p-IGF1R, p-Akt, and p-ERK is due to the cytotoxic effects of deguelin, SCC-4 cells were exposed to different concentrations of deguelin for 24 h and then examined for cell viability by trypan blue dye-exclusion method. Cell viability remained about 90% at 10 *μ*M or less for 24 h and it decreased by 60% at 100 *μ*M (Figure 4(a)). Since a decrease in p-IGF1R, p-Akt, and p-ERK was seen in the cells 24 h after deguelin treatment at either 1.0 or 10 *μ*M (see Figures 4(a) and 4(b)), it was suggested that deguelin-mediated decreases in p-IGF1R, p-Akt, and p-ERK levels are not due to its cytotoxic effects.

### 3.5. Inhibition of p-Akt rather than Inhibition of p-ERK Is Associated with Deguelin-Induced Apoptosis in SCC-4 Cell Line

**Figure 5:**
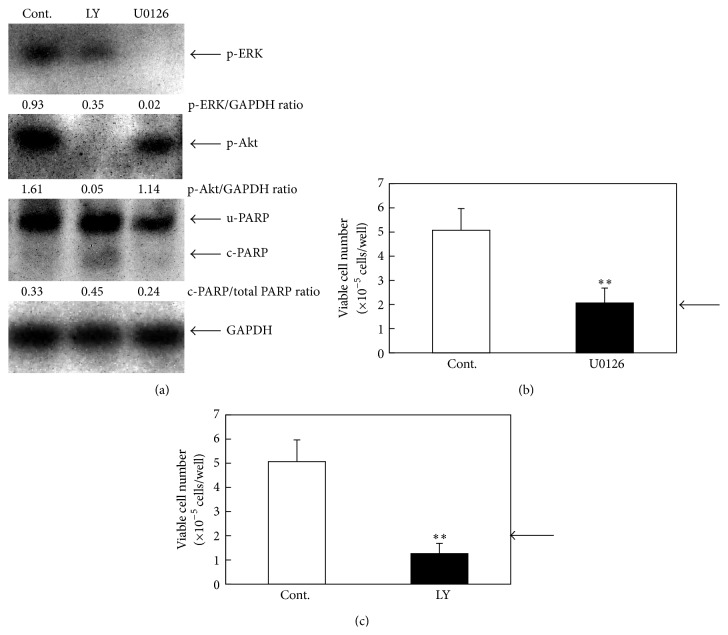
Inhibition of activated Akt rather than inhibition of activated ERK is associated with deguelin-induced apoptosis in SCC-4 cells. (a) Subconfluent culture was incubated for 24 h in serum-free medium. After the starvation, cells were treated with U0126 (10 *μ*M) or LY294002 (50 *μ*M) for 1 h, and cells were incubated for 15 min in 10% FBS-containing medium. Whole-cell lysates were extracted and analyzed by Western blot using antibodies against p-Akt, p-ERK, and PARP (c-PARP, cleaved PARP; u-PARP, uncleaved PARP; total PARP, sum of cleaved and uncleaved PARP). Trypan-blue dye exclusion assay was performed to measure cell viability of SCC-4 cells at 24 h after U0126 (10 *μ*M) (b) or LY294002 (50 *μ*M) (c) treatment. Arrows indicate inoculated cell numbers. Each point represents the mean ± SD from triplicate assay (^∗∗^ *P* < 0.01).

As general understanding, Akt signaling and ERK signaling are important as survival and proliferation, respectively. In addition, in fibroblast cells, ERK signaling is considered to be survival signal [19]. Therefore, in order to confirm that the apoptotic effect of deguelin is mediated by interacting with Akt signaling or ERK signaling in SCC-4 cells, we examined the effects of ERK inhibitor U0126 and PI-3 kinase/Akt inhibitor LY294002. As expected, U0126 inhibited phosphorylation of ERK while it did not affect PARP cleavage (Figure 5(a)). Furthermore, U0126 suppressed the proliferation of SCC-4 cells without any cytotoxicity because viable cell number after U0126 treatment remained unchanged with the vehicle control (Figure 5(b)). On the contrary, LY294002 reduced p-Akt while it cleaved PARP (Figure 5(a)). LY294002 also suppressed the cell viability of SCC-4 and viable cell number after LY294002 treatment was less than the vehicle control (Figure 5(c)). These results strongly suggest the involvement of the inhibition of the PI-3 kinase/Akt pathway rather than the inhibition of the MEK/ERK pathway in the deguelin-induced apoptosis.

### 3.6. Deguelin Induced Apoptosis by Reducing IGF-Stimulated Akt Activation in SCC-4 Cells

**Figure 6:**
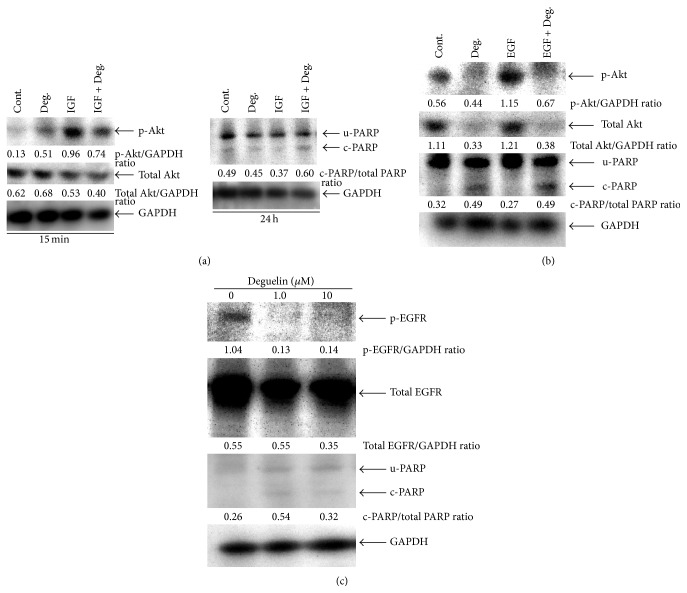
Deguelin induced apoptosis by targeting both EGFR-Akt and IGF1R-Akt pathways in HNSCC cell lines. Subconfluent cultures were incubated for 24 h in serum-free medium. After the starvation, cells were treated with 10 *μ*M deguelin for 1 h. (a) The deguelin-treated SCC-4 cells were incubated for 15 min and 24 h with or without 10 ng/ml of IGF, respectively. (b) The deguelin-treated HSC-4 cells were incubated for 24 h with or without 10 ng/ml of EGF. Whole-cell extracts were analyzed by Western blot using antibodies against p-Akt, Akt, and PARP. (c) HSC-4 cells were treated with deguelin at different concentrations for 24 h in 10% FBS-containing medium. Whole-cell extracts were analyzed by Western blot using antibodies against p-EGFR, EGFR, and PARP (c-PARP, cleaved PARP; u-PARP, uncleaved PARP; total PARP, sum of cleaved and uncleaved PARP).

Next, we examined whether deguelin induced apoptosis by reducing IGF1-Akt signaling in SCC-4 cells. As shown in Figure 6(a), p-Akt was elevated by IGF1 treatment for 15 min and this induction was suppressed by deguelin accompanied with increase in the cleaved PARP. These results clearly indicated that deguelin induced apoptosis by targeting IGF1R-Akt pathway in SCC-4 cells.

### 3.7. Deguelin Induced Apoptosis Accompanied with the Reduction of Constitutive and EGF-Stimulated Akt Activation in HSC-4 Cell Line

Finally, we examined whether deguelin induced apoptosis accompanied with the reduction of constitutive and EGF-stimulated Akt activation in HSC-4 cells. As shown in Figure 6(b), deguelin increased in the levels of cleaved-PARP accompanied with the reduction of both constitutive and EGF-stimulated p-Akt protein levels. Furthermore, deguelin induced apoptosis by reducing p-EGFR expression in HSC-4 cells, as shown in Figure 6(c). These results clearly suggested that deguelin induced apoptosis by targeting EGFR-Akt pathway in HSC-4 cells.

## 4. Discussion

We showed that deguelin induced cell death in HNSCC cell lines. To better understand the action mechanisms of deguelin, we further examined intracellular signaling. We found that deguelin induced apoptosis by targeting IGF1R-Akt and targeting EGFR-Akt pathways in HNSCC cell lines. To the best of our knowledge, this is the first report that deguelin can target both EGFR-Akt and IGF1R-Akt pathways in HNSCC cell lines. Previously, deguelin was reported to induce apoptosis by autophagy through AMPK-Ulk signaling, inhibition of Akt signaling, and degradation of CDK4/Survivin in HNSCC [15]. Another report indicated that deguelin suppressed NF-*κ*B in SCC-4 cells [20]. Therefore, many signaling pathways may work together to exert the antitumor effect of deguelin, and our studies extended the fact that deguelin has an applicable potential for HNSCC therapy.

Inhibition of activated Akt rather than inhibition of activated ERK is associated with deguelin-induced apoptosis in HNSCC. Recent study has suggested crosstalk between Akt signaling and ERK signaling: for example, feedback from the PI3K-Akt-mTORC1 (mammalian target of rapamycin complex 1) to the Ras-MEK-ERK pathway [21] and ERK activates Akt signaling at the mTOR level [22]. However, in SCC-4 cells, we indicated that inhibition of activated Akt rather than inhibition of activated ERK is associated with deguelin-induced apoptosis because U0126 showed cytostatic effect without changes of PARP cleavage level and LY294002 had cytotoxic effect with increase in PARP cleavage. Probably, crosstalk between two signalings seems to be cell type specific.

Deguelin was proposed as an inhibitor of Hsp90 [23]. The client protein of HSP 90 includes Akt, EGFR, and IGF1R. EGFR is expressed at high levels in the majority of epithelial malignancies including HNSCC [6]. Elevated expression of EGFR in HNSCC correlates with poor prognosis, and EGFR has been a target of anticancer treatments due to its critical roles in cell survival and proliferation [7]. Therefore, cetuximab, antibody of EGFR, is an applicable strategy for HNSCC therapy [24]. However, Jameson et al. [25] postulated that IGF1R-Akt signaling underlies cetuximab resistance for HNSCC. Therefore, deguelin should be applicable for HNSCC as combination with EGFR inhibitors such as cetuximab and erlotinib.

## 5. Conclusion

Deguelin possessed antitumor effect in HNSCC by targeting both EGFR-Akt and IGF1R-Akt pathways. Because deguelin is reported to be nontoxic and tolerable in the animal model [26], deguelin should be an applicable strategy for HNSCC therapy.
